# Modulation of mitochondrial DNA copy number in a model of glioblastoma induces changes to DNA methylation and gene expression of the nuclear genome in tumours

**DOI:** 10.1186/s13072-018-0223-z

**Published:** 2018-09-12

**Authors:** Xin Sun, Justin C. St John

**Affiliations:** 1 Mitochondrial Genetics Group, Hudson Institute of Medical Research, 27-31 Wright Street, Clayton, VIC 3168 Australia; 2 Department of Molecular and Translational Sciences, Monash University, 27-31 Wright Street, Clayton, VIC 3168 Australia

**Keywords:** Mitochondrial DNA, DNA methylation, Gene expression, Tumorigenesis, POLG

## Abstract

**Background:**

There are multiple copies of mitochondrial DNA (mtDNA) present in each cell type, and they are strictly regulated in a cell-specific manner by a group of nuclear-encoded mtDNA-specific replication factors. This strict regulation of mtDNA copy number is mediated by cell-specific DNA methylation of these replication factors. Glioblastoma multiforme, HSR-GBM1, cells are hyper-methylated and maintain low mtDNA copy number to support their tumorigenic status. We have previously shown that when HSR-GBM1 cells with 50% of their original mtDNA content were inoculated into mice, tumours grew more aggressively than non-depleted cells. However, when the cells possessed only 3% and 0.2% of their original mtDNA content, tumour formation was less frequent and the initiation of tumorigenesis was significantly delayed. Importantly, the process of tumorigenesis was dependent on mtDNA copy number being restored to pre-depletion levels.

**Results:**

By performing whole genome MeDIP-Seq and RNA-Seq on tumours generated from cells possessing 100%, 50%, 0.3% and 0.2% of their original mtDNA content, we determined that restoration of mtDNA copy number caused significant changes to both the nuclear methylome and its transcriptome for each tumour type. The affected genes were specifically associated with gene networks and pathways involving behaviour, nervous system development, cell differentiation and regulation of transcription and cellular processes. The mtDNA-specific replication factors were also modulated.

**Conclusions:**

Our results highlight the bidirectional control of the nuclear and mitochondrial genomes through modulation of DNA methylation to control mtDNA copy number, which, in turn, modulates nuclear gene expression during tumorigenesis.

**Electronic supplementary material:**

The online version of this article (10.1186/s13072-018-0223-z) contains supplementary material, which is available to authorized users.

## Background

Mammalian cells have multiple copies of the mitochondrial genome (mtDNA) within each mitochondrion. The human mitochondrial genome is a circular, double-stranded genome that is 16.6 kb in size. It is essential for the production of cellular ATP, as it encodes 13 subunits of the electron transfer chain that conducts oxidative phosphorylation (OXPHOS) and is the cell’s major generator of ATP. It also encodes 22 transfer RNAs and 2 ribosomal RNAs [1].

mtDNA replication is driven by nuclear-encoded mtDNA-specific replication factors that translocate to the mitochondrion. The primary factor is the mtDNA-specific polymerase, polymerase gamma [2, 3], which is a heterotrimer enzyme composed of a catalytic subunit, subunit A (POLG), and two supporting subunits, subunits B (POLG2) [4]. The process of mtDNA replication is also supported by the mitochondrial helicase twinkle (TWNK), the single-stranded DNA binding protein (SSBP1) and the mitochondrial topoisomerase (TOP1MT) [5–7]. There are several other key factors including mitochondrial transcription factors A (TFAM), B1 (TFB1M) and B2 (TFB2M), which generate the precursor transcript used to initiate mtDNA replication [8, 9]. Furthermore, some upstream regulators of these mtDNA replication factors are highly involved in mitochondrial biogenesis and function. These include nuclear respiratory factors 1 and 2 (NRF1/2), peroxisome proliferator-activated receptor γ (PPARG) and its co-activator PGC1α (PPARGC1A), the Sirtuin family of genes (SIRT1-3), and the oestrogen-related receptors (ESRRA/B/G) [10–14].

mtDNA replication is strictly regulated during development and differentiation, which enables mature cells to acquire the requisite numbers of mtDNA copy to support their specific functions [15–18]. Initially, this is achieved by pluripotent (naïve) cells having established the mtDNA set point (reviewed in [19]). These naïve cells each possess around 200 copies of mtDNA, which promotes glycolysis as the favoured form of energy production, and cellular proliferation [20]. These copies of mtDNA serve as the initial template for mtDNA replication, allowing cells to acquire the appropriate numbers of mtDNA copy as they differentiate into mature cell types [16–18, 20, 21].

The inability to regulate mtDNA copy number affects cellular function and impedes developmental potential. For example, oocytes at the metaphase II stage that possess too few copies of mtDNA frequently fail to fertilize [22–25]. Likewise, certain types of cancer cells exhibit low mtDNA copy number and are unable to successfully complete differentiation [16, 18]. Indeed, cancer cells mainly rely on aerobic glycolysis for energy production, which allows for higher rates of cellular proliferation and prevents differentiation from taking place [16]. However, when they are induced to differentiate, they appear to be trapped in a ‘pseudo-differentiated’ state as they had not previously maintained the mtDNA set point and, as a result, the nuclear and mitochondrial genomes do not act in synchrony [19, 26]. Interestingly, when cancer cells are partially depleted of their mtDNA content, they undergo dedifferentiation and are then able to replicate mtDNA as they undergo differentiation, suggesting that they have re-established the mtDNA set point and synchrony between the nuclear and mitochondrial genomes [18].

During the early stages of development, the nuclear genome undergoes DNA demethylation mediated by the ten-eleven translocation methylcytosine dioxygenases (TET enzymes) to erase parental DNA methylation profiles. De novo DNA methylation then takes place to establish a new DNA methylation profile directed by the DNA methyltransferase family (DNMTs), which primes naïve cells for cellular differentiation [27, 28]. To this extent, cell-specific gene expression profiles are established during differentiation in synchrony with changes to DNA methylation patterns. Likewise, the DNA methylation status at exon 2 of *POLG* is a determinant of when this gene is expressed and, in turn, regulates mtDNA copy number in a cell-specific manner [15, 16]. This is supported by experiments using DNA demethylation agents, such as 5-azacytidine [29] and vitamin C [30], where modulation of DNA methylation at exon 2 of *POLG* increased mtDNA copy number in HSR-GBM1 cells derived from a glioblastoma multiforme (GBM) tumour [16, 18, 31].

The HSR-GBM1 cell line is a high-grade malignant GBM cell line that is characterized as being similar to stem-like neural precursors and is extensively DNA methylated, which contributes to its tumorigenic gene profile [32, 33]. However, its hyper-methylated profile is not established by the overexpression of the isocitrate dehydrogenases (IDH1/2) that harbour onco-mutations, as the alleles for these genes are wild type [34, 35]. Under normal circumstances, IDH enzymes act on the citric acid cycle to generate α-ketoglutarate, which is a co-factor of the TET enzymes that modulate DNA demethylation patterns [36–40]. However, overexpression of and mutations to the *IDH* genes in GBM result in a metabolic switch that produces 2-hydroxyglutarate and restricts DNA demethylation induced by the TET enzymes [36–40]. Consequently, HSR-GBM1 cells enable the analysis of modifications to DNA methylation profiles to be undertaken whereby the DNA methylation status of the cells is not influenced by mutations to key regulators of DNA demethylation and thus allows the effects of mtDNA copy number to be studied independently of these influences.

Interestingly, mtDNA depletion of HSR-GBM1 cells to varying amounts of mtDNA copy number affected tumour progression and frequency when these cells were inoculated into mice [18]. Progression and frequency were greatest in cells depleted to 50% of their original content, but tumour formation was less frequent and took significantly longer when cells possessed only 3% and 0.2% of their original mtDNA content [18]. Notably, mtDNA copy was restored to similar levels during in vivo tumorigenesis accompanied by DNA demethylation at exon 2 of *POLG* [17].

In order to determine whether global DNA methylation profiles were modulated following the restoration and maintenance of mtDNA copy number in end point tumours, we investigated the DNA methylation profiles of GBM tumours derived from HSR-GBM1 cells that possessed varying amounts of mtDNA copy number and exhibited different frequencies and progression in tumour formation. We used whole genome methylated DNA immunoprecipitation (MeDIP)-Seq. We matched the modulated regions with their transcriptional profiles to focus on their effects on gene expression. We also investigated the mtDNA replication factors to determine how they responded to the newly established interactions between the nucleus and the mitochondrial genome. Our results highlight the bidirectional control of the nuclear and mitochondrial genomes through modulation of DNA methylation to control mtDNA copy number and gene expression in tumours using the HSR-GBM1 cell line as a model.

## Results

### Replenishment of mtDNA copy number

**Fig. 1:**
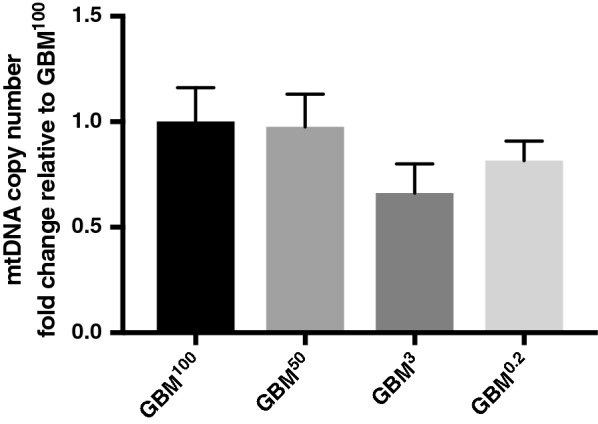
Replenishment of mtDNA copy number in the GBM^50^, GBM^3^ and GBM^0.2^ tumours. Fold change in mtDNA copy number for GBM^50^, GBM^3^ and GBM^0.2^ tumours relative to the GBM^100^ tumours. GBM^100^, GBM^50^, GBM^3^ and GBM^0.2^ tumours were harvested at 65 days, 61 days, 83 days and 90 days after inoculation, respectively. Statistical significance was determined using one-way ANOVA (*n* = 3 tumours per group; technical triplicates per sample). No significant differences were identified between the groups

Tumours were previously generated from HSR-GBM1 cells possessing different levels of mtDNA copy number, namely GBM^100^ (possessing 100% of their mtDNA content), GBM^50^ (50% mtDNA content), GBM^3^ (3% mtDNA content) and GBM^0.2^ (0.2% mtDNA content) cells [18]. Tumours arising from GBM^3^ and GBM^0.2^ cells had significantly delayed initiation and reached end point at 83 and 90 days, respectively. They had a lower frequency of tumour formation (6/12 and 2/12, respectively) compared with GBM^100^ tumours, which took 65 days to reach end point with tumours forming from 11/12 inoculations [18]. GBM^50^ tumours had accelerated formation (61 days) [18]. Furthermore, mtDNA copy number was replenished in each of the end point tumours to pre-depletion levels without significant differences (Fig. 1), which highlights the need for sufficient mtDNA copy number to promote tumorigenesis, and likely explains why delayed tumour formation was observed in cells with lower levels of mtDNA copy number.

### Modifications to DNA methylation resulting from re-establishment of mtDNA copy number during tumorigenesis

In order to determine whether DNA methylation of the nuclear genome was modulated following re-establishment of mtDNA copy number during tumour progression, we assessed the global DNA methylation profiles of each of the tumour types at end point. Whole genome MeDIP-Seq was performed on DNA extracted from individual tumours from each cohort to identify key regions that might be modulated. Using the MEDIPS package, the quality of the sequencing reads was determined using the saturation and enrichment analyses and each achieved high standards, indicating that there was sufficient coverage and enrichment of the methylated DNA (Additional file 1). Regions with total coverage of 100 × (minrowsum = 100) or greater reads were used for statistical analysis, which is deemed to be a more stringent filter than the default threshold of tenfold coverage [31].

A common feature of cancer cells is genomic instability, which causes significant copy number variation (CNV) when compared to healthy controls [41]. CNV can also affect the outcomes from comparative analysis after MeDIP-Seq, which, if ignored, can introduce false positive results due to CNVs inducing higher or lower levels of enrichment rather than being reflective of the real differences in DNA methylation [41]. Using the Illumina Global Screening Array, no CNVs were identified to be significantly different between the tumour groups, as assessed using the NEXUS Copy Number Module. This negates the potential effect of CNV on the analysis of MeDIP-Seq outcomes (see Additional file 2).

Differentially methylated regions (DMRs) were identified between the GBM^100^ tumours and tumours formed from cells possessing varying levels of mtDNA. Table 1 shows the number of DMRs identified in the GBM^50^, GBM^3^ and GBM^0.2^ tumours when compared with the GBM^100^ tumours using different filters for statistical significance. With the increase in statistical power (*p* value) from ≤ 0.05, 0.01 to 0.001, fewer DMRs were identified in each comparison. Overall, there were fewer DMRs identified in the GBM^3^ tumours followed by the GBM^50^ tumours whilst there were far more DMRs identified in the GBM^0.2^ tumours. Using a cut-off *p* value of 0.001, for instance, 124 DMRs were identified between the GBM^50^ tumours and the GBM^100^ tumours, 36 DMRs for the GBM^3^ tumours and 16,565 DMRs for the GBM^0.2^ tumours. The greater number of DMRs identified for the GBM^0.2^ tumours indicated that the DNA methylation profile for this cohort of tumours was extensively modified as a result of the increased levels of mtDNA depletion in their initiating cells and subsequent restoration during tumorigenesis. We further applied the Bonferroni correction to adjust *p* values to control for false positive calls. After correction, there were 143 DMRs identified in the GBM^0.2^ tumours, but there were no significant DMRs identified in the GBM^50^ and GBM^3^ cohorts of tumours.

**Table 1:** The number of DMRs identified in tumours (*n* = 3 per group) formed from depleted cells compared with non-depleted cells

Comparisons	*p* ≤ 0.05	*p* ≤ 0.01	*p* ≤ 0.001	Adj. *p* ≤ 0.05
GBM^50^ versus GBM^100^	8980 (16.85%: 83.15%)	1135 (10.57%: 89.43%)	124 (7.26%: 92.74%)	–
GBM^3^ versus GBM^100^	4940 (34.76%: 65.24%)	597 (41.88%: 58.12%)	36 (44.44%: 55.56%)	–
GBM^0.2^ versus GBM^100^	51,366 (11.25%: 88.75%)	34,060 (3.66%: 96.34%)	16,565 (2.14%: 97.86%)	143 (21.68%: 78.32%)

The ratio of hyper- and hypo-methylated DMRs (expressed as percentages) is shown in brackets

Both hyper- and hypo-methylated states were observed for tumours derived from cells with lower levels of mtDNA copy number. However, the majority of the DMRs were hypo-methylated compared to the GBM^100^ tumours (Table 1). There were 31 hyper-methylated DMRs (21.68%) identified in the comparison between GBM^0.2^ and GBM^100^ tumours using an adjusted *p* value of 0.05. Indeed, the tendency to hypo-methylation was also observed in the other tumours. Taking the results identified using a *p* value of, for instance, 0.001, only 7.26% of the DMRs in the GBM^50^ tumours and 44.44% of the DMRs in the GBM^3^ tumours were hyper-methylated. The different proportions of hypo- and hyper-methylation further suggest that when tumorigenesis was initiated from different levels of mtDNA copy number, different patterns of DNA methylation were induced.

### The annotation of the DMRs and gene ontology analysis for the DMR-overlapping genes

**Fig. 2:**
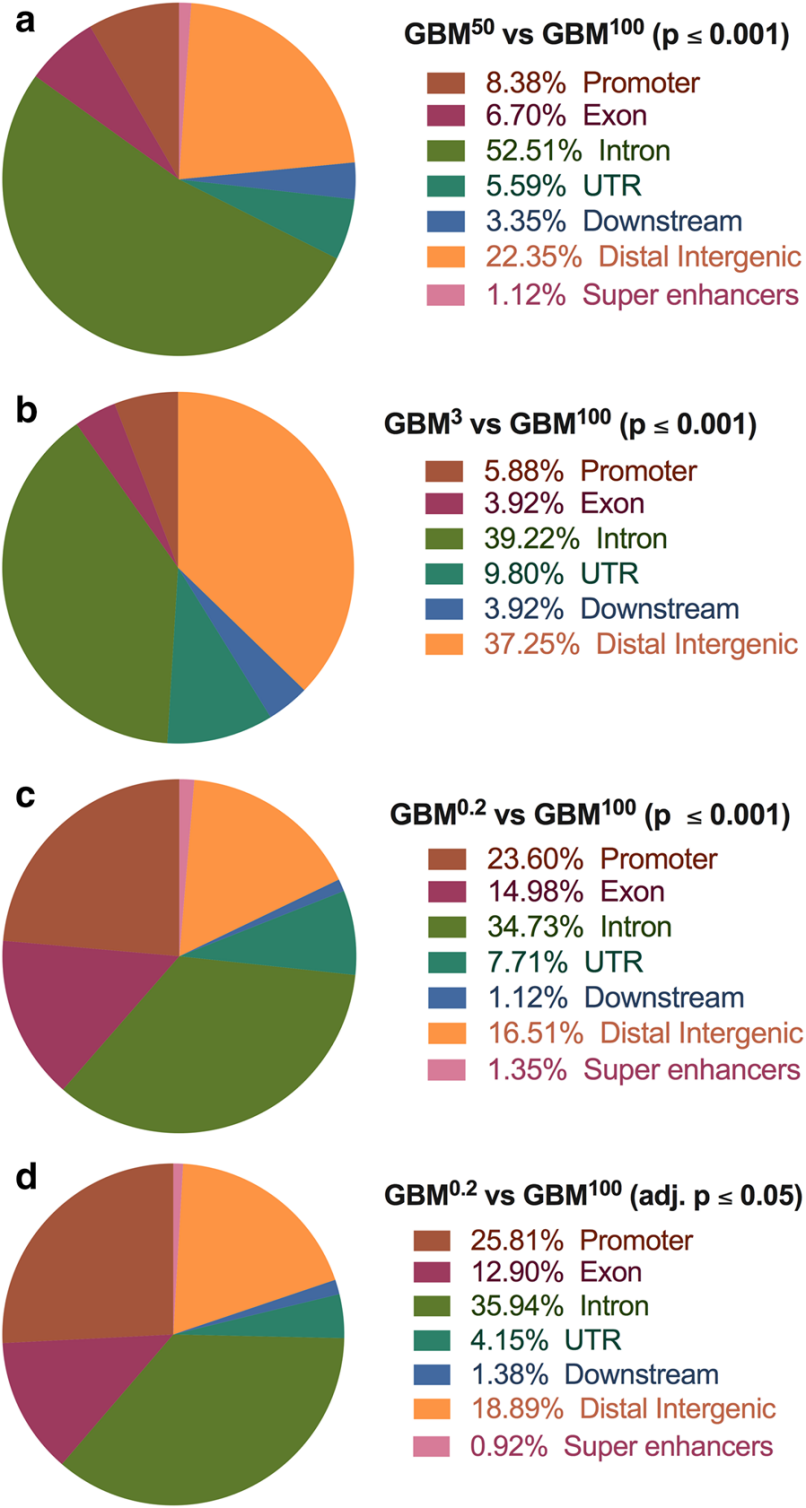
Annotations of the DMRs. Based on their genomic locations, DMRs identified in the comparison: **a** GBM^50^ versus GBM^100^ (*p* ≤ 0.001); **b** GBM^3^ versus GBM^100^ (*p* ≤ 0.001); c GBM^0.2^ versus GBM^100^ (*p* ≤ 0.001); and **d** GBM^0.2^ versus GBM^100^ (adjusted. *p* ≤ 0.05) were classified into promoters, exons, introns, UTRs, downstream regions, distal intergenic regions and super enhancers using the ChIPSeeker package (*n* = 3 tumours per group). DMR-overlapping regions in brown signify promoter regions, violet-red indicates exons, green indicates introns, blue indicates promoters, teal represents UTRs, blue represents downstream regions, orange indicates intergenic regions, and pink indicates super enhancers. The percentage of each group is indicated in the key to each figure

Firstly, the annotation of genomic regions was performed on the four groups of DMRs identified using the filters of a *p* value ≤ 0.001 and an adjusted *p* value ≤ 0.05 [GBM^50^ vs GBM^100^ (*p* ≤ 0.001), GBM^3^ vs GBM^100^ (*p* ≤ 0.001), GBM^0.2^ vs GBM^100^ (*p* ≤ 0.001) and GBM^0.2^ vs GBM^100^ (adjusted *p* ≤ 0.05)]. Overall, the results showed that over 49% of the DMRs were enriched in intragenic regions, namely exons, introns and promoter regions, and they were the most enriched regions (Fig. 2a–d). Each of these four groups of DMRs showed the greatest enrichment at introns, which was 52.51% of the GBM^50^ DMRs (Fig. 2a), 39.22% of the GBM^3^ DMRs (Fig. 2b), 34.73% of the GBM^0.2^ DMRs (Fig. 2c; *p* ≤ 0.001) and 35.94% of the GBM^0.2^ DMRs (Fig. 2d; adjusted *p* ≤ 0.05). For the GBM^50^ and GBM^3^ DMRs, the second most enriched regions were the distal intergenic regions, which were enriched at 22.35% and 37.25%, respectively (Fig. 2a, b). Interestingly, for the GBM^0.2^ DMRs, identified using the filters of *p* ≤ 0.001 and adjusted *p* ≤ 0.05, similar patterns were observed regardless of the numbers of DMRs (Fig. 2c, d). In contrast to the GBM^50^ and GBM^3^ DMRs, the second most enriched region for the GBM^0.2^ DMRs was the promoter regions, which were 23.60% and 25.81% of the DMRs after filtering at *p* ≤ 0.001 and adjusted *p* ≤ 0.05, respectively (Fig. 2c, d). The distal intergenic regions were ranked third accounting for 16.51% and 18.89% of the GBM^0.2^ DMRs identified using the filters of *p* ≤ 0.001 and adjusted *p* ≤ 0.05, respectively (Fig. 2c, d). Untranslated regions (UTRs), downstream regions of genes and super enhancers accounted for less than 14% of the DMRs in each comparison. Interestingly, over 80% of the identified DMRs overlapped with topologically associating domains (TADs) that interact with other genomic regions and are considered to be the regulatory units of the genome [42].

**Fig. 3:**
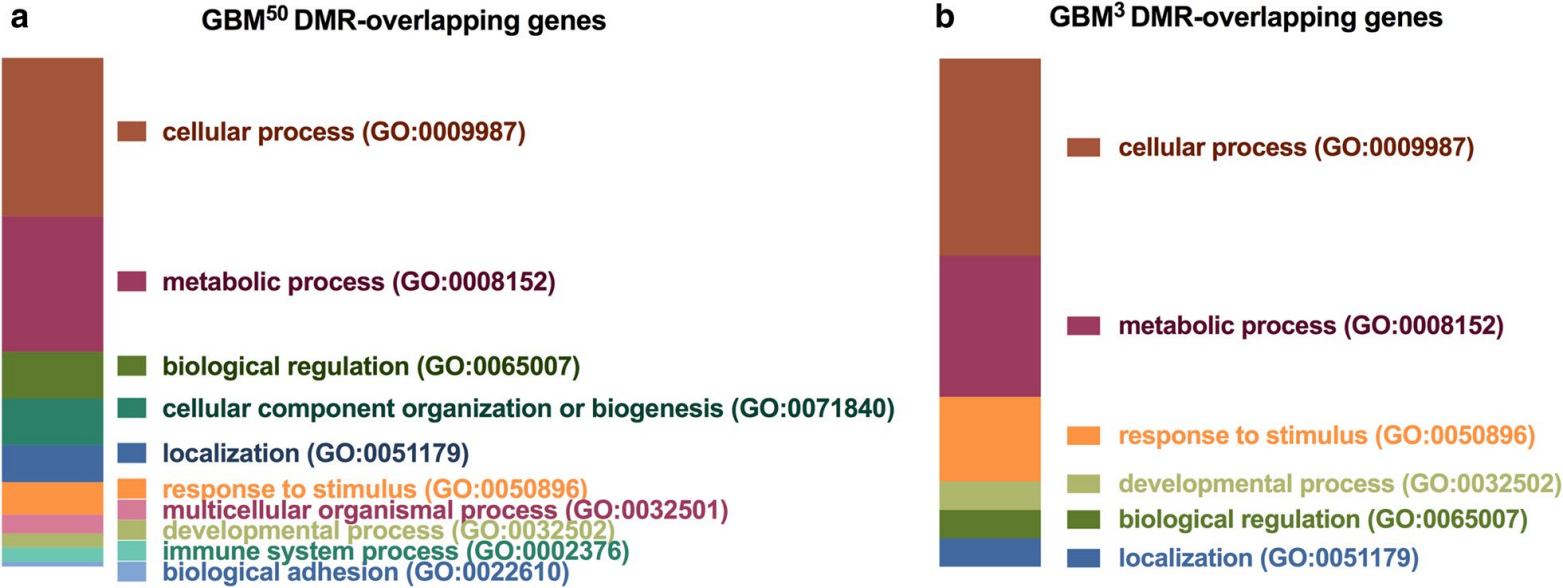
Functional classification analysis of the DMR-overlapping genes in the GBM^50^ and GBM^3^ tumours. DMR-overlapping genes in: **a** GBM^50^ tumours; and **b** GBM^3^ tumours were classified into subgroups based on their associated biological processes using the PANTHER system

To understand the related functions of the DMRs, functional classification and statistical overrepresentation analyses were performed on the DMR-overlapping genes using the PANTHER Classification System. The functional classification of the DMR-overlapping genes showed that the GBM^50^ and GBM^3^ tumours mostly clustered into cellular and metabolic processes (Fig. 3). Furthermore, the pathways of biological regulation, localization, response to stimuli and developmental processes were present in both cohorts (Fig. 3). Due to the large number of DMR-overlapping genes identified in the GBM^0.2^ tumours using the two different filters, statistical overrepresentation analysis was, therefore, performed. Interestingly, the biological processes enriched in the DMR-overlapping genes showed strong associations with developmental processes, especially in the DMR-overlapping genes identified with an adjusted *p* value of 0.05 (Table 2). The common developmental pathways identified in both analyses (shown in italics) included developmental, nervous system development and cell differentiation processes (Table 2). This is in line with a previous finding that, after partial mtDNA depletion with 7 and 14-day ddC treatment, differentiation was able to take place in the GBM cells [18]. Moreover, regulation of transcription from the RNA polymerase II promoter was found to be commonly modulated, which reflected the potential for transcriptional changes that could result from the DMRs.

**Table 2:** Pathways affected by the DMR-overlapping genes in the GBM^0.2^ tumours using the PANTHER Classification system

PANTHER pathways	Input/background	Fold enrichment	*p* value	FDR	PANTHER pathways	Input/background	Fold enrichment	*p* value	FDR
DMR-overlapping genes in the GBM^0.2^ tumours (*p* ≤ 0.001)					DMR-overlapping genes in the GBM^0.2^ tumours (adj. *p* ≤ 0.05)				
* Cell differentiation (GO:0030154)*	191/548	1.47	6.12E−06	9.95E−05	* Cell differentiation (GO:0030154)*	9/459	3.93	5.42E−04	4.41E−02
* Nervous system development (GO:0007399)*	113/314	1.52	1.88E−04	1.76E−03	* Nervous system Development (GO:0007399)*	7/238	5.89	2.23E−04	5.45E−02
* Regulation of transcription from RNA polymerase II promoter (GO:0006357)*	196/598	1.39	9.47E−05	9.63E−04	* Regulation of transcription from RNA polymerase II promoter (GO:0006357)*	10/548	3.66	4.61E−04	5.63E−02
					Cellular process (GO:0009987)	57/7905	1.45	5.54E−04	2.70E−02
					Behaviour (GO:0007610)	4/76	10.55	6.79E−04	2.76E−02
Developmental process (GO:0032502)	537/1501	1.51	1.01E−16	2.46E−14					
Sensory perception of smell (GO:0007608)	6/240	0.11	1.41E−15	1.72E−13					
Defence response to bacterium (GO:0042742)	0/112	< 0.01	9.10E−11	4.44E−09					
Response to biotic stimulus (GO:0009607)	3/144	0.09	2.98E−10	1.21E−08					
B cell mediated immunity (GO:0019724)	0/94	< 0.01	3.08E−09	9.40E−08					
Complement activation (GO:0006956)	1/94	0.04	5.91E−08	1.44E−06					
Ectoderm development (GO:0007398)	96/212	1.91	3.82E−07	8.46E−06					
Cell recognition (GO:0008037)	4/105	0.16	3.64E−06	6.84E−05					
Mesoderm development (GO:0007498)	109/269	1.71	4.74E−06	8.27E−05					
Phagocytosis (GO:0006909)	15/182	0.35	9.91E−06	1.42E−04					
Embryo development (GO:0009790)	49/106	1.95	1.96E−04	1.77E−03					
Cellular component morphogenesis (GO:0032989)	144/423	1.44	2.32E−04	2.02E−03					
Biological adhesion (GO:0022610)	124/356	1.47	2.72E−04	2.21E−03					
Cell adhesion (GO:0007155)	124/356	1.47	2.72E−04	2.29E−03					
Segment specification (GO:0007379)	37/78	2.01	7.86E−04	5.99E−03					
Transmembrane receptor protein tyrosine kinase signalling pathway (GO:0007169)	60/151	1.68	1.07E−03	7.90E−03					
Negative regulation of apoptotic process (GO:0043066)	43/99	1.84	1.25E−03	8.73E−03					
Regulation of phosphate metabolic process (GO:0019220)	169/537	1.33	1.34E−03	8.86E−03					
Intracellular signal transduction (GO:0035556)	310/1071	1.22	1.55E−03	9.92E−03					
Anterior/posterior axis specification (GO:0009948)	12/16	3.17	3.37E−03	1.96E−02					
Cytoskeleton organization (GO:0007010)	129/404	1.35	3.74E−03	2.12E−02					
Synaptic transmission (GO:0007268)	121/382	1.34	5.92E−03	3.28E−02					

Common processes are highlighted in italics

### Validation of the gene expression of the regulators of DNA methylation

**Fig. 4:**
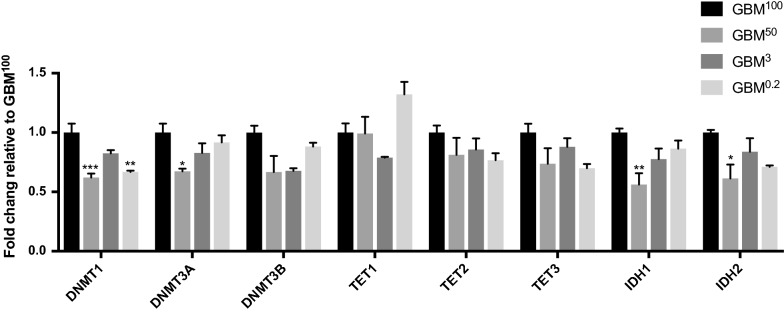
Differential expression of the regulators of DNA methylation. Bars represent the mean of the relative quantification levels relative to the GBM^100^ tumours (relative expression = 1). Error bars show SEM (*n* = 3 tumours per group; technical replicates per sample). Statistical significance was determined by One-way ANOVA. *, ** and *** *p* values < 0.05, 0.01 and 0.001, respectively

DNA methyltransferases (DNMTs), namely DNMT1/3A/3B, are responsible for catalysing DNA methylation at cytosines by converting them to 5-methylcytosines (5mC). On the other hand, the ten-eleven translocation (TET) enzymes, namely TET1/2/3, can demethylate 5mC to 5-hydroxymethylcytosine (5hmC) [27, 28]. The changes to these DNA methylation and demethylation factors can provide insights into how the changes to the DNA methylation profiles occurred. Therefore, the gene expression levels of the DNA methylation regulators were assessed using a custom-made Fluidigm qPCR array (Fig. 4). There was ≥ 1.5-fold change in expression of *DNMT1* in GBM^50^ and GBM^0.2^ tumours, suggesting that the reduction in mtDNA copy number strongly induced DNA demethylation in the tumours, and is probably the major reason why the majority of regions were hypo-methylated (Table 1). *DNMT3A* was also significantly down-regulated by over 1.5-fold in the GBM^50^ tumours (Fig. 4). None of the DNA methylation factors was found to be differentially expressed in the GBM^3^ tumours compared with the GBM^100^ tumours, which further supported the results that fewer DMRs were identified in this group. The expression of *TET1* showed a trend of up-regulation only in the GBM^0.2^ tumours. As there was a higher percentage of hyper-DMRs in the GBM^0.2^ tumours, the up-regulation of *TET1* might be a response to counter the increased hyper-methylation observed.

Furthermore, it has been reported that the genomes of low grade and secondary glioblastomas are extensively methylated to support the tumorigenic transcriptome that they exhibit [32, 38–40, 43]. This is further supported by the findings that the overexpression of the mutated isocitrate dehydrogenase (IDH1/2) in the citric acid cycle inhibits the activity of TET2 and thus contributes to the hyper-methylated genome [36–40]. However, the HSR-GBM cell line possesses wild-type alleles for *IDH1/2* [34, 35], which makes it an excellent model to investigate the impact of mtDNA content in tumour-initiating cells on DNA methylation patterns during tumorigenesis, especially as non-mutated *IDH1* has been shown to promote cell growth in GBM [44]. We, therefore, investigated the expression of *IDH1/2* in these tumours and found that expression for both genes was down-regulated in the GBM^50^ tumours (Fig. 4), which likely reduced their ability to induce hyper-methylation and supports the hypo-methylation observed in these tumours.

### DMR-overlapping differentially expressed genes and differentiation markers after restoration of tumorigenic capacity

**Fig. 5:**
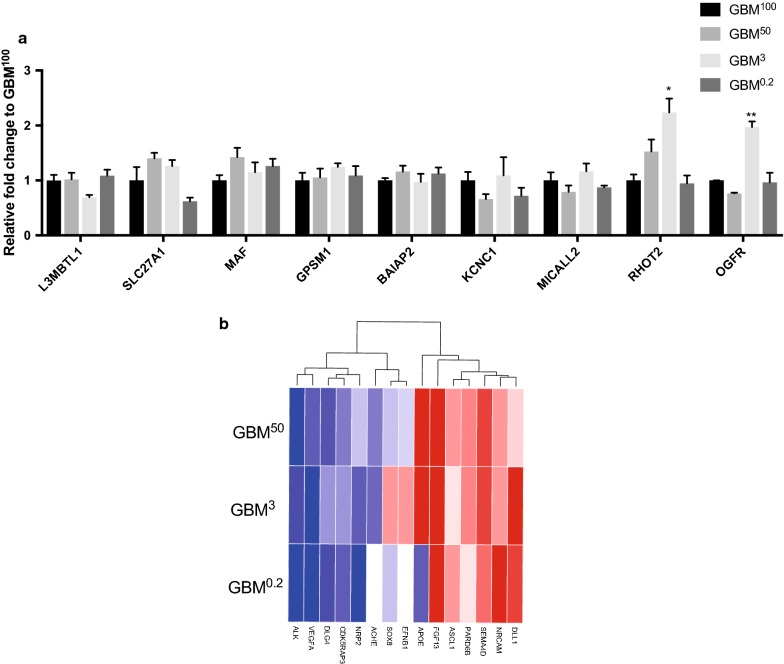
Transcriptional profiles of DMR-overlapping differentially expressed genes and markers of neurogenesis and neural stem cells. **a** Differential expression of the DMR-overlapping differentially expressed genes. Bars represent the mean of the relative quantification levels relative to the GBM^100^ tumours (relative expression = 1). Statistical significance was determined by One-way ANOVA. Error bars show SEM (*n* = 3 per group; technical triplicates per sample). *, ** *p* values < 0.05 and 0.01, respectively. **b** Heatmap of markers of neurogenesis and neural stem cells from the RNA-Seq profiles. For the GBM^50^, GBM^3^ and GBM^0.2^ cohorts, the fold changes (log2) of read counts to the mean value of the GBM^100^ cohort were plotted. The colour scheme from blue, white to red represents the level of expression from twofold down-regulation to twofold up-regulation

To investigate which genes changed their patterns of expression in response to the altered levels of DNA methylation, we compared the identified DMRs with the differentially expressed genes previously reported on the same samples [17]. We focused on the DMRs identified using an adjusted *p* value of 0.05, which were considered to be the genomic regions that had undergone most significant changes in DNA methylation. There were 9 DMR-overlapping differentially expressed genes, namely *BAIAP2*, *L3MBT1L*, *KCNC1*, *GPSM1*, *SLC27A1*, *MAF*, *OGFR*, *MICALL2* and *RHOT2* (Fig. 5a). The changes to the levels of gene expression were further validated for these genes (Fig. 5a). Interestingly, the GBM^3^ tumours, with the least number of DMRs, showed more variable patterns in expression amongst the DMR-overlapping genes when compared with the GBM^100^ tumours. *RHOT2* and *OGFR* were significantly up-regulated in the GBM^3^ tumours (*p* < 0.05 and *p* < 0.01, respectively). The greater modification that occurred in the DNA methylation profiles of the GBM^50^ and GBM^0.2^ tumours promoted gene expression profiles more similar to the tumorigenic features of the GBM^100^ tumours, whereas less modification in the GBM^3^ tumours failed to mimic the transcriptional changes in these DMR-overlapping genes.

As the DMRs were found to be tightly associated with developmental processes (Fig. 3 and Table 2), we also focused on the neural markers and genes in neurogenesis that have been reported in cells having undergone mtDNA depletion to the same levels as those used to form tumours [18]. Indeed, it has been previously shown that the partial mtDNA depletion of GBM cells could lead to increased expression of genes associated with early developmental processes [18]. In tumours formed from these cells, an overview of gene expression identified by RNA-Seq data is shown (Fig. 5b). Both up-regulation and down-regulation were observed. To make a comparison between the in vivo tumours and in vitro cells, the negative regulator of cell proliferation *ALK* was found to be down-regulated in the mtDNA-depleted tumours. However, *ALK* was found to be up-regulated in the mtDNA-depleted cultured cells. Likewise, the regulators of cell differentiation and proliferation, *VEGFA, DLG4, CDK, NRP2* and *ACHE*, were also found to be down-regulated in the mtDNA-depleted tumours but presented higher levels of expression in the cells. The regulators of transcription and differentiation, *ASCL1* and *DLL1* in the Notch signalling pathway, were found to be up-regulated in the mtDNA-depleted tumours but down-regulated in the cells. The regulator of synaptogenesis, *APOE*, was found to be up-regulated in the GBM^50^ and GBM^3^ tumours, but down-regulated in the GBM^0.2^ tumours and the mtDNA-depleted cells. Moreover, *FGF13, SEMA4D, PAPD6B and NRCAM* were found to be commonly up-regulated in both cells and tumours. As the cells recovered their tumorigenicity after restoration of mtDNA copy number in vivo, the shifts in gene expression between the tumours and the cells indicated that the restoration and maintenance of mtDNA copy number in tumorigenesis is tightly associated with the regulation of differentiation.

### Modulation of the mtDNA replication factors for the maintenance of mtDNA copy number in tumorigenesis

**Fig. 6:**
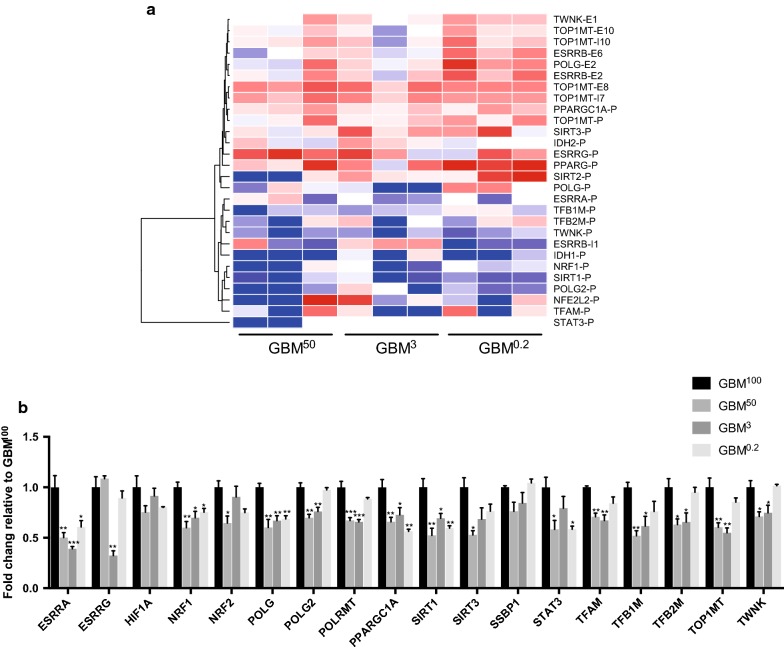
Overview of the changes to DNA methylation and transcription of the mtDNA replication factors. **a** DNA methylation levels of the intragenic CGIs associated with the mtDNA replication factors. Fold changes in relative methylation scores to the GBM^100^ tumours were plotted for the three biological replicates from the GBM^50^, GBM^3^ and GBM^0.2^ cohorts. The colour scheme from blue, white to red represents the level of DNA methylation from low to high. “P”, “E” and “I” following each gene name indicates CGIs that are located at the promoter regions, exons and introns, respectively. **b** Differential expression of the mtDNA replication factors. Bars represent the mean of the relative quantification levels relative to the GBM^100^ tumours (relative expression = 1). Error bars show SEM (*n* = 3 tumours per group; technical replicates per sample). Statistical significance was determined by one-way ANOVA. *, ** and *** *p* values < 0.05, 0.01 and 0.001, respectively

As mtDNA copy number was replenished to similar levels in each of the tumour types (Fig. 1), we determined how the mtDNA-specific replication factors were affected. In total, 20 known mtDNA replication factors were screened for differentially methylated intragenic CpG islands (CGIs). The fold changes in relative methylation scores for the GBM^100^ tumours of 28 promoter and gene-body CGIs within the mtDNA replication factors were plotted (Fig. 6a). An increasing trend in fold changes was observed from the GBM^50^ to the GBM^0.2^ tumours. Particularly, for the CGIs, higher levels of methylation were found in the GBM^50^ tumours compared to the GBM^100^ tumours (shown in red blocks) and the fold changes generally increased to an even higher level in the GBM^0.2^ tumours. This trend was mostly observed amongst the gene-body CGIs. For the CGIs that were more hypo-methylated in the GBM^50^ tumours than the GBM^100^ tumours (shown in blue blocks), the levels of DNA demethylation were indicative of minor shifts in the GBM^0.2^ tumours.

Furthermore, the gene expression levels were also assessed using a Fluidigm qPCR array (Fig. 6b). Overall, the levels of expression of the mtDNA replication factors in the tumours formed from cells possessing varying levels of mtDNA were found to be down-regulated, which likely restricts mtDNA replication during tumorigenesis after the restoration of mtDNA copy number to its original levels. *ESRRA, NRF1, POLG*, *PPARGCA1* and *SIRT1* were identified to be significantly down-regulated in all of the mtDNA-depleted tumours. The mtDNA replication factors *POLG2, TOP1* *MT* and *TWNK* were also found to be down-regulated in the GBM^50^ and GBM^3^ tumours. Moreover, the key mtDNA transcription factors *POLRMT, TFAM, TFB1M* and *TFB2M* were down-regulated in the GBM^50^ and GBM^3^ tumours. *NRF2* and *SIRT3* were only down-regulated in the GBM^50^ tumours. The hypoxia regulator *STAT3* was found to be significantly down-regulated in the GBM^50^ and GBM^0.2^ tumours*. ESRRG* was only down-regulated in the GBM^3^ tumours. This indicates that mtDNA replenishment had been completed and the low levels of expression of the mtDNA replication factors were indicative of mtDNA turnover during cell division rather than the active repopulation of depleted cells with mtDNA.

## Discussion

We have shown that bidirectional cooperation between the nuclear and mitochondrial genomes is important for tumour formation. To this extent, the DNA methylation and gene expression profiles of the nuclear genome were extensively modulated following the restoration of mtDNA copy number to pre-depletion levels in tumours that developed to end point. Whilst it is conceivable that the mtDNA depletion process did not reduce mtDNA copy in individual cells from each group uniformly, it is unlikely that this occurred. If a small population of cells with higher mtDNA copy number had been selected for, there would have been greater similarity amongst the DNA methylation and gene expression profiles across the groups. However, cells from each group exhibited distinct gene expression profiles prior to inoculation [18] and DNA methylation and gene expression profiles as end point tumours.

The restoration of mtDNA copy number can be achieved through the cell’s own mtDNA replication machinery or the horizontal transfer of mitochondria from surrounding cells [17, 45, 46]. It was previously shown that the same tumours possessed only human mtDNA and not mouse mtDNA from the surrounding stroma [47]. However, the restoration of mtDNA copy number also involved the accumulation of mtDNA variants, which increased in number as a function of the degree of restoration of mtDNA copy number. Each of the tumours exhibited a gain in de novo variants primarily associated with *ND4* and *ND6*, which encode subunits of complex I of the electron transfer chain, and the D-loop. However, these variants have been identified in other GBM cell lines [47]. Consequently, the presence and modulation of mtDNA are important to tumorigenesis given that cells require mtDNA to be fully functional [48].

The effects of DNA methylation on transcription vary dependent on the location of the methylated sites. Methylated CGIs within the promoter regions are known to repress transcription [49], whereas methylation within gene bodies correlates positively and negatively with transcriptional elongation [50]. In our work, the degree of restoration of mtDNA copy number differentially affected the levels of DNA methylation in the end point tumours. However, the majority of the DMRs were hypo-methylated in each comparison. Whilst there was high enrichment in the intragenic regions, namely promoters, exons and introns, there were differences in the numbers of DMRs affected and their distribution across the nuclear genome, especially for the promoter and exon regions amongst the comparisons. The DMRs in the GBM^50^ and GBM^0.2^ tumours also overlapped with several enhancers, which are documented to contribute to tumour progression and cancer cell plasticity [51]. Furthermore, over 80% of the DMRs overlapped with TADs, the regulatory units of the genome [42], which suggests that their modulation could affect the structural formation and, therefore, genetic activities of the genome [42].

These outcomes are likely mediated by the down-regulation in levels of expression of the DNA methyltransferases, especially *DNMT1*. For the GBM^0.2^ tumours, DNA demethylation was further enhanced by the up-regulation of *TET1*, which mediates the transition from 5mC to 5hmC [52], resulting in these tumours being most hypo-methylated. *IDH1* and *IDH2*, the cytoplasmic and mitochondrial isoforms respectively, had lower levels of expression in the tumours derived from mtDNA-depleted cells than GBM^100^ tumours. This suggests that α-ketoglutarate could continue to act as a co-factor of the TET enzymes and did not induce a switch to 2-hydroxyglutarate to promote hyper-methylation [43, 44, 53]. This is consistent with HSR-GBM1 cells possessing wild type copies of the IDH genes [34, 35], which is common in high-grade (IV) astrocytomas [38, 40, 44]. One-carbon metabolism also takes place in the mitochondria resulting in the generation of S-adenosylmethionine, the universal methyl group donor [54]. S-adenosylmethionine is present at abnormal levels in cancer cells and could affect DNA methylation profiles during tumorigenesis [54]. We would anticipate that S-adenosylmethionine activity was down-regulated as a result of mtDNA depletion, which reduces mitochondrial function [55], and, thus, promotes the hypo-methylated state of the resultant tumours.

**Fig. 7:**
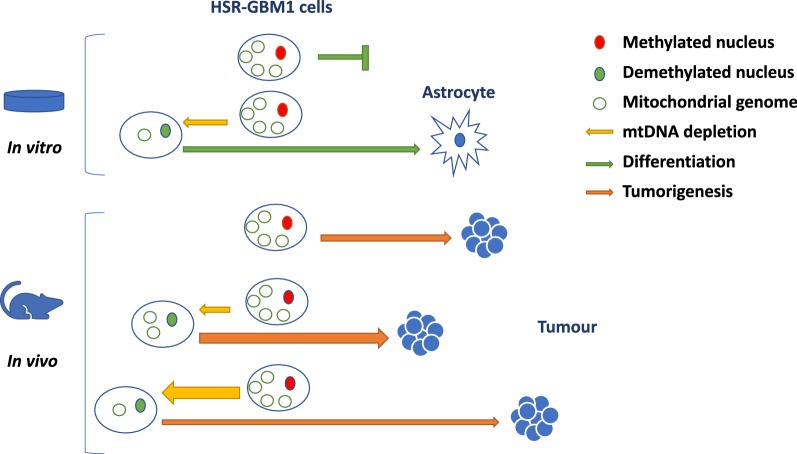
Modulation of mtDNA copy number affects the fate of HSR-GBM1 cells. In the in vitro model of HSR-GBM1 cells, depletion of mtDNA (yellow arrow) for 7/14 days results in demethylation of the nuclear genome (green) and rescues differentiation to astrocytes that is otherwise blocked (green arrow). In the in vivo model of HSR-GBM1 cells, varying levels of depletion result in different frequencies of progression to tumorigenesis (orange arrow). 50% depletion accelerated tumorigenesis (thicker orange arrow), whereas further depletion slowed the initiation of tumorigenesis by modulating differentiation networks, nuclear gene expression and the mtDNA replication factors

During early development, large-scale DNA demethylation takes place to reset the nuclear genome to a naïve state [27, 28]. This is coupled with the strict regulation of mtDNA copy number to establish the mtDNA set point [15]. As a result, the two genomes act in tandem to overcome a number of molecular check points at different stages of development, which include mtDNA replication turnover events [56]. Indeed, *Polg*-*/*- mice do not possess sufficient mtDNA at E6.5 and cannot initiate mtDNA replication at this key developmental check point and, consequently, die [57]. Likewise, somatic cells reprogrammed to a naïve, pluripotent state, similar to embryonic stem cells, can fail to re-establish the mtDNA set point and lose their differentiation potential [56]. HSR-GBM1 cells are cancer stem cells that express key early neural markers, maintain low mtDNA copy number and use glycolysis for energy production, which are typical facets of a stem cell [33]. However, when they are induced to differentiate into high OXPHOS-derived ATP requiring cells, for example neurons and astrocytes, they fail to replicate mtDNA in synchrony with changes to the nuclear genome and their progress stalls [56]. Nevertheless, mtDNA partially depleted HSR-GBM1 cells can re-establish their mtDNA set point and expand their mtDNA copy number in order to complete differentiation [18] (please refer to Fig. 7 ‘in vitro’). However, the degree of restoration of mtDNA copy number influenced the differentiation and transcriptional potentials of the cells as determined by RNA-Seq analysis of end point tumours. The most affected networks between the GBM^100^ and the GBM^0.2^ and GBM^3^ tumours were cellular development, cellular growth and proliferation, whilst cancer, cell cycle and cellular development were most affected in the GBM^50^ tumours [17].

Although the GBM^50^ tumours are more aggressive, they appear to restrict mtDNA replenishment to pre-depletion levels, which ensures that the balance between mtDNA copy number and the methylation of the chromosomal genome is at levels that promote tumorigenesis, as is the case for the GBM^3^ and GBM^0.2^ tumours. From a mtDNA perspective, there is increased hyper-methylation in the majority of gene-body CGIs of the mtDNA replication factors of the mtDNA-depleted tumours compared with the GBM^100^ tumours. This is exemplified by increased methylation at exon 2 of *POLG* and exon 8 of *TOP1MT* and decreased levels of expression for these two genes, which are known to regulate mtDNA copy number in a cell-specific manner through DNA methylation at these intragenic regions [15, 16, 31]. Interestingly, the use of DNA methylation agents, such as 5-Azacytidine and Vitamin C, on cultured HSR-GBM1 and reprogrammed somatic cells can restore mtDNA replication turnover events and promote differentiation [16], which likely explains the use of these agents in clinical settings for cancer patients [58, 59].

The DMR-overlapping genes affected a variety of biological processes. In the GBM^50^ and GBM^3^ tumours, the primary effects were on cellular and metabolic processes. In the GBM^0.2^ tumours, developmental processes were more overrepresented, including cell differentiation and nervous system development. Of the nine DMR-overlapping differentially expressed genes, four were specific to neurogenesis. *SLC27A1* was significantly down-regulated in a *SOX2*-knockdown GBM cell line characterized by abolished dedifferentiation and decreased tumorigenesis [60]. Mutations in *KCNC1* have been reported as the driving cause for progressive myoclonus epilepsies [61]. Indeed, a number of DNA methylation signatures have been identified in several neurodevelopmental syndromes, including Coffin–Siris, Kabuki and CHARGE syndromes, which are implicated in a variety of cancers [62, 63].

Although the GBM^3^ tumours presented with the least number of DMRs, they had more variable patterns of expression amongst the DMR-overlapping genes. For example, *RHOT2* and *OGFR* were significantly up-regulated in the GBM^3^ tumours. RHOT2 is a miro GTPase that regulates mitochondrial transport, distribution and dynamics, especially in neurodegenerative disorders [64, 65], and is differentially expressed in different types (CD133^+^ and CD133^−^) of GBM stem cells [66]. It is usually only expressed in type I GBM cancer stem cells and is co-expressed with the stem cell markers *SOX2, SOX11* and *OLIG2* [66]. This indicates the expression of *RHOT2* is important for regulating mitochondria and their dynamics during early development, which requires the presence of mtDNA given that cells need mtDNA to be fully functional [48]. On the other hand, *OGFR* is a negative regulator of cell proliferation and reduces proliferation of astrocytes in cell culture [67]. Consequently, GBM^3^ tumours differentially expressed two genes associated with different stages of development unlike the other tumour types. Nevertheless, the greater levels of modification that occurred in the DNA methylation profiles of the GBM^50^ and GBM^0.2^ tumours promoted gene expression profiles more indicative of the tumorigenic features of the GBM^100^ tumours. A similar situation has been observed in tumour cell lines possessing the same chromosomal background but different mtDNA haplotypes where early tumours from each haplotype expressed a distinct set of genes [68]. However, in this case, a series of commonly expressed genes were expressed again highlighting how the mitochondrial genome can affect tumorigenesis.

It is evident that GBM^0.2^ cells, which had undergone a longer period of mtDNA depletion through an agent that specifically targets POLG from interacting with the mitochondrial genome [69], adopted a more dedifferentiated state than the partially depleted cell types, as demonstrated by the expression of early neural developmental genes [18]. Indeed, many key regulators of cell proliferation and differentiation were down-regulated in the mtDNA-depleted tumours, but up-regulated in the mtDNA-depleted cells [18], whilst regulators of transcription and differentiation in the Notch signalling pathway were up-regulated in the tumours derived from mtDNA-depleted cells but down-regulated in the cells [18]. Likewise, the regulator of synaptogenesis, *APOE*, was up-regulated in the GBM^50^ and GBM^3^ tumours but down-regulated in the mtDNA-depleted cells [18]. Consequently, since the mtDNA-depleted cells recovered their tumorigenicity as mtDNA copy number was restored in vivo, it is evident that the restoration and maintenance of mtDNA copy number in tumour-initiating cells is tightly associated with the regulation of neural differentiation in glioblastoma.

The regulation of mtDNA copy number, DNA methylation and nuclear gene expression at different stages of development highlights the need for cells to establish a balance between these three components to promote their specific fates. In the case of tumours derived from cells with different levels of mtDNA, unique DNA methylation profiles are established that regulate gene expression and mtDNA copy number in end point tumours (see Fig. 7 ‘in vivo’). To this extent, GBM^0.2^ cells attempted to modulate global DNA methylation patterns and transcription of the mtDNA replication factors and differentiation markers at the expense of tumorigenesis resulting in fewer tumours forming and requiring significantly longer to form. However, GBM^50^ cells were more tumorigenic, as they had struck the appropriate balance between mtDNA copy number and the methylated state of the chromosomal genome to promote tumorigenesis. The proposed model shown in Fig. 7 indicates how modulation of mtDNA copy number in tumour-initiating cells induces changes to DNA methylation of the nuclear genome and, therefore, can affect the fate of tumour cells in in vitro and in vivo environments. This highlights the synergy required between the two genomes in establishing tumorigenesis.

## Conclusions

In all, we have shown that the restoration of mtDNA copy number during tumorigenesis induces major changes to the nuclear genome that resulted in differential DNA methylation and expression of genes. These changes enriched developmental processes and essential metabolic pathways associated with GBM. In addition, the changes to the nuclear-encoded mtDNA replication factors highlight the synergy between the nuclear and mitochondrial genomes in restoring tumorigenic capacity. This was clearly demonstrated in the GBM^0.2^ tumours formed from cells having undergone the highest levels of depletion and requiring the longest to initiate tumour formation. They underwent more extensive DNA methylation remodelling at key CGIs and within the intragenic regions of the mtDNA replication factors to maintain similar transcriptional levels. Whilst we recognize that we have only focused on the HSR-GBM1 tumour model, our results highlight the bidirectional control of the nuclear and mitochondrial genomes through modulation of DNA methylation in response to mtDNA copy number and to control gene expression in tumorigenesis.

## Methods

### Cell culture and Xenograft models

GBM tumours were previously generated from HSR-GBM1 cells possessing different levels of mtDNA copy number after treatment with 10 μm 2′–3′-dideoxycytidine (ddC), a mtDNA depletion agent that directly inhibits the interaction of POLG with the mitochondrial genome [69], in the presence of 50 mg/mL uridine (Sigma-Aldrich, MO, USA). Cells were depleted to 50% (GBM^50^), 3% (GBM^3^), 0.2% (GBM^0.2^), and 100% (GBM^100^) of their original mtDNA content, as described in [18]. To this extent, HSR-GBM1 cells were cultured in complete neural stem cell media consisting of Dulbecco’s Modified Eagle Medium/Nutrient Mixture (DMEM/F-12) Media (Thermo Fisher Scientific, MA, USA), 2% StemPro neural supplement (Thermo Fisher Scientific), 20 ng/mL basic fibroblast growth factor (bFGF; Merck Millipore MO, USA) and 20 ng/mL epidermal growth factor (EGF; Merck Millipore) at 37 °C, 5% CO_2_ and 95% humidity.

The animal work was approved by the Animal Ethics Committee, Monash University, Approval Number: MMCA/2011/76. Briefly, 0.5 million HSR-GBM1 tumour cells in 100 mL of medium were inoculated subcutaneously into both flanks of 5- to 6-week-old, female BALB/c nude mice (Animal Research Centre, Perth, Australia). Tumour growth rates and volumes were reported in [18]. For each group, cells were injected into 12 mice to form tumours, as detailed in [18]. In all, 11 tumours formed from GBM^100^ cells, 10 from GBM^50^ cells, 6 from GBM^3^ cells and 2 from GBM^0.2^ cells [18]. GBM^100^ tumours reached an average volume of 175 mm^3^ at 65 days, GBM^50^ tumours reached an average volume of 250 mm^3^ at 61 days (*p* > 0.05), GBM^3^ tumours reached an average volume of ~ 150 mm^3^ at 83 days (*p* < 0.01), and GBM^0.2^ tumours reached an average volume of > 200 mm^3^ at 90 days (*p* < 0.01) [18].

### DNA extraction

Total genomic DNA was extracted from the tumours using the DNeasy Blood & Tissue Kits (Qiagen, CA, USA), according to manufacturer’s protocols with minor modifications. The DNA samples were treated with 3 μL of RNase solution (Qiagen) at room temperature. DNA samples were eluted in 100 μL of autoclaved Milli-Q H_2_O.

### Determination of mtDNA copy number per cell

mtDNA copy number per cell was determined, as previously described [16]. To this extent, concentrations of qPCR products for *β*-*globin* and mtDNA were determined against standard curves generated by quantitative real-time PCR (qPCR; Rotor-Gene 3000, Corbett Research, Cambridge, UK) on total DNA purified from the tumours (*n* = 3 per group; *n* = 3 replicates per sample). Primer sequences and primer-specific reaction conditions are listed in Additional file 3. mtDNA copy number per cell was calculated using the formula of 2 × *N*_mtDNA_*/N*_β-globin_, where, for *N*_mtDNA_ and *N*_β-globin_, *N* = (qPCR product concentration × 6.023 × 10^14^)/(qPCR product size in bp × 660).

### Immunoprecipitation of methylated DNA sequencing (MeDIP-Seq)

Five microgram of genomic DNA from each of the tumour samples (*n* = 3 per group) underwent MeDIP, as previously described [70]. Briefly, each DNA sample was sheared into 200–1000 bp using the Covaris Adaptive Focused Acoustics (AFA™) S220 system (Woburn, MA, USA). dsDNA was then denatured to single-stranded DNA, as required for the antibody, by incubation at 95 °C for 10 min. 3 μg of each DNA sample was immunoprecipitated with 2 μg of anti-5mC antibody (Active Motif) with 20 μL per sample of prewashed Dynabeads^®^ Protein G (Thermo Fisher Scientific). The suspension was incubated in 500 μL of IP buffer (100 mM sodium phosphate (pH 7.0); 1.4 M NaCl;0.5% Triton X-100) at 4 °C for 16 h under rotation. The beads were then washed three times with 1 mL of IP buffer and resuspended in 250 μL of proteinase K digestion buffer (50 mM Tris-HCl, pH 8.0; 10 M EDTA, pH 8.0; 1.0% SDS) with 10 μL of proteinase K (20 mg/mL; Bioline, London, UK). The suspension was incubated at 50 °C for 3 h on a thermo-shaker. The supernatant was then collected on a magnetic particle concentrator (Thermo Fisher Scientific). DNA was purified from the supernatant using the QIAquick PCR Purification Kit (Qiagen), according to the manufacturer’s protocol.

MeDIP products from each of the tumour samples underwent library construction using the DNA SMART ChIP-Seq Kit (Clontech, CA, USA), as per the manufacturer’s protocol (No. 021115). Libraries were quality checked using Qubit fluorometric quantitation (Thermo Fisher Scientific), a 2100 Bioanalyzer (Agilent, CA, USA), and qPCR. All libraries were of a similar size (297–335 bp) and quantities (~ 40 ng/μl). A single equimolar pool was made following denaturation of the libraries at 94 °C for 2 min. 12 pM of each library was used for onboard cluster generation and sequencing in 4 lanes of two consecutive Illumina HiSeq 1500 Rapid runs. Cluster densities were within the optimal range of 881–959 k/mm^2^ (optimal 750–1000 k/mm^2^). This resulted in around 170 million readable clusters per lane. 95% of the clusters passed filter, and 96% of the reads passed the Illumina sequencing quality score of Q30, which was deemed excellent quality for base-calling. Run quality parameters with the PhiX spike-in had an error rate of 0.12% (expected < 0.5%) and phasing/pre-phasing of < 0.13/< 0.10 (expected < 0.4/< 0.2).

### Computational analysis for MeDIP-Seq

#### Mapping

Computational analysis was performed on the MeDIP-Seq sequences using the customized analytical pipeline, as described in [31]. Briefly, the sequences were firstly checked for quality and adaptors using the FastQC software (v 0.11.5). The raw sequences were then aligned to the human reference genome GRCh38/hg38 (UCSC) using the Burrows-Wheeler Aligner (BWA) software (version 0.7.16a) in the single-end mode of the BWA-backtrack algorithm with default settings [71]. The output files (*.sam) were then combined and converted to bam files (*.bam) by SAMtools (version 1.4).

#### Determination of differentially methylated regions (DMRs)

Uniquely mapped reads with mapping scores (MAPQ) ≥ 30 were kept for analytical analysis using the MEDIPS package (version 1.24.0), according to the command codes of the MEDIPS package. Briefly, the reads were cleaned for PCR duplicates that mapped to exactly the same genomic positions by setting the parameter “uniq” to 1. The reads were then extended to 500 nucleotides according to the reference genome without any shift in the genomic locations by setting the parameter “extend” to 500 and “shift” to 0. The coverage of every 100-bp window of the genome was summarized by setting the parameter “window_size” to 100. MeDIP-Seq-specific quality control analysis was performed on each sample, which includes saturation analysis and CpG enrichment analysis. A coupling factor was set up based on the GBM^100^ sample for normalization. Differentially methylated windows (DMWs) with the total count of reads ≥ 100 (minrowsum = 100) were identified between groups using the ‘edgeR’ method. Statistically significant DMWs were selected for downstream analysis. Continuous significant DMWs were merged as one DMR (Additional file 4).

#### Annotation of DMRs

DMRs then underwent annotation based on their corresponding genomic regions in the human genome using the ChIPSeeker package (version 3.5) [72]. Super-enhancer annotation was performed using the dbSUPER databases [73]. TAD overlapping analysis was conducted using the TAD datasets available in The ENCODE project [74]. Gene lists were analysed for gene ontology using the Panther Classification System (version 12.0) [75]. The functional classification analysis was used to annotate the functions of genes. The statistical overrepresentation test (Fisher’s exact test with a false discovery rate (FDR) correction) was used to identify the significance of associated biological processes overrepresented amongst the genes.

#### Region-of-Interest (ROI) analysis

DNA methylation levels at particular regions of the genome, for example CGIs associated with the mtDNA replication factors, were assessed by the built-in ROI analysis in the MEDIPS package. DNA methylation levels of ROIs were expressed as relative methylation scores, as determined by the MEDIPS package. The relative methylation score was developed specifically for MeDIP-Seq in order to normalize methylation scores for regions based on the concept of CpG coupling analysis [76, 77].

### Determination of copy number variation (CNV)

Total DNA from the tumour samples (*n* = 3 per group) was submitted to the Australian Genome Research Facility (AGRF; VIC, AUS) to perform genotyping. The Illumina Human Global Screening Array Beadchip (Illumina) was used, which covers approximately 700 K SNPs throughout the genome. Array data were normalized, clustered and underwent genotype calling using the Genotype Module of GenomeStudio 2.0 (Illumina), according to the user’s manual. The full data report containing log R ratios and B allele frequencies for each probe was then exported and analysed using Nexus 9.0 software (BioDiscovery Inc., CA, USA). The Nexus Copy Number Module was used to identify regions of CN gain and loss, loss of heterozygosity (LOH) for each group and statistically significant CNVs between groups (*p* value ≤ 0.05). Human genome hg19 was chosen as the reference genome to annotate the genomic locations. Genomic regions were then aligned to human genome hg38, allowing overlapping analysis with the DMRs using the ‘intersect’ function of bedtools (v2.24.0) [78, 79].

### Identification of DMR-overlapping differentially expressed genes

RNA-Seq was previously performed on the GBM tumours [17]. The sequencing files were deposited in the NCBI Sequence Read Archive (SRA) under the accession code PRJNA296542 [17]. The sequences were mapped to the human genome (hg19) using the Tophat aligner (v1.3.1) [17, 80]. Differentially expressed genes were determined using the Cufflinks tool (v2.2.1) [17, 80]. Differentially expressed genes (FDR ≤ 0.05) underwent lift-over analysis to match the hg38 human genome assembly using the GALAXY platform (usegalaxy.org) and then overlapping analysis was performed with the DMRs using the ‘intersect’ function of bedtools (v2.24.0) [78, 79].

### Gene expression analysis using real-time quantitative PCR

Total genomic RNA was extracted from the tumour samples (*n* = 3 per group) using the RNeasy Mini Kit and the QIAshredder (Qiagen, CA, USA), according to manufacturer’s protocols with minor modifications. The RNA samples were treated with DNase I (Qiagen) on column for 20 min. cDNA was synthesized from 1 μg of the total RNA using the Superscript III First-Strand synthesis system (Thermo Fisher Scientific), according to the manufacturer’s instructions.

cDNA products (*n* = 3 technical replicates per tumour) were assessed using the Rotor-Gene 3000 RT-PCR machine under primer-specific conditions (Additional file 3), as described in [16]. Relative gene expression to the GBM^100^ tumours was calculated using the ΔΔCT method. *OAZ1, 18SrRNA* and *HPRT1* were used as the housekeeping genes, of which the mean values of expression were used as the internal control for data normalization [31]. Data were represented as the fold change to the GBM^100^ group (*n* = 3; mean ± SEM). One-way ANOVA was used to determine statistical significance between the GBM^50^, GBM^3^ and GBM^0.2^ tumours and the GBM^100^ tumours. Results were plotted using GraphPad Prism 7 (GraphPad Software, Inc., CA, USA).

### Gene expression analysis using the Fluidigm platform

Using the same sets of cDNA samples (*n* = 3 per group; *n* = 2 replicates per sample), gene expression of targets of interest was assessed using the Fluidigm qPCR array, according to the manufacturer’s instructions. Taqman primers are listed in Additional file 5. Taqman primers were pooled and diluted in C1 DNA suspension buffer to a final concentration for each primer of 180 nM. Each cDNA sample and a non-template control underwent pre-amplification for 14 cycles, according to the manufacturer’s instructions (Quick Reference PN 100-5876 B1). The pre-amplification reaction consisted of the Taqman PreAmp Master mix (Thermo Fisher Scientific) and the pooled Taqman primers. Products were diluted fivefold with C1 DNA suspension buffer. The integrated fluidic circuit controller HX was then used to prime and load the 96.96 Dynamic array plate. 5 μL of each sample was loaded in duplicate into each sample inlet and 5 μL of each Taqman assay (10 x) were loaded into each assay inlet. Real-time qPCR was performed according to the Biomark GE 96.96 Standard v2 protocol. Data were exported using the Fluidigm real-time PCR analysis software (v4.1.1). Relative gene expression was calculated using the same method described above.

## Additional files

**Additional file 1.** MeDIP-Seq specific QC results determined by the MEDIPS package.

**Additional file 2.** Summary of CNV regions identified in each cohort of tumours and overlapping with DMRs using the Nexus 9.0 software.

**Additional file 3.** Primer pairs for real time PCR.

**Additional file 4.** DMRs identified using MeDIP-Seq and MEDIPS package.

**Additional file 5.** Taqman assays used in the Fluidigm qPCR arrays.

## Abbreviations

mtDNA: mitochondrial DNA
OXPHOS: oxidative phosphorylation
POLG: polymerase gamma subunit A
POLG2: polymerase gamma subunit B
TWNK: mitochondrial helicase
SSBP1: single-stranded DNA binding protein
TOP1MT: mitochondrial topoisomerase
TFAM: mitochondrial transcription factors A
TFB1M: mitochondrial transcription factors B1
TFB2M: mitochondrial transcription factors B2
NRF1/2: nuclear respiratory factors 1/2
PPARG: peroxisome proliferator-activated receptor γ
PPARGC1A: peroxisome proliferator-activated receptor γ co-activator
SIRT1-3: Sirtuin family of genes 1-3
ESRRA/B/G: oestrogen-related receptors A/B/G
TET: ten-eleven translocation methylcytosine dioxygenases
DNMT: DNA methyltransferase family
GBM: glioblastoma multiforme
MeDIP: methylated DNA immunoprecipitation
GBM^100^: tumours formed from cells possessing 100% of their mtDNA content
GBM^50^: tumours formed from cells possessing 50% of their mtDNA content
GBM^3^: tumours formed from cells possessing 3% of mtDNA content
GBM^0.2^: tumours formed from cells possessing 0.2% of mtDNA content
DMW: differentially methylated windows
DMR: differentially methylated regions
ROI: region of interest
CNV: copy number variation
5mC: 5-methylcytosine
5hmC: 5-hydroxymethylcytosine
CGI: CpG island
TADs: topologically associating domains
UTR: untranslated regions
ddC: 2′-3′-dideoxycytidine
BWA: Burrows-Wheeler Aligner
FDR: false discovery rate
LOH: loss of heterozygosity
