# Supplementary material for: Modulation of mitochondrial DNA copy number in a model of glioblastoma induces changes to DNA methylation and gene expression of the nuclear genome in tumours
Source: Epigenetics Chromatin. 2018 Sep 12;11:53. doi: 10.1186/s13072-018-0223-z (PMC6136172; doi:10.1186/s13072-018-0223-z)
Supplement: Supplementary file 1 — Additional file 1. MeDIP-Seq specific QC results determined by the MEDIPS package. [file 13072_2018_223_MOESM1_ESM.docx]

**Additional file 1. MeDIP-Seq specific QC results determined by the MEDIPS package**.

|  | **Saturation Score** | **Enrichment Score** |
| --- | --- | --- |
| **GBM^100^-1** | 0.97 | 2.07 |
| **GBM^100^-2** | 0.98 | 2.55 |
| **GBM^100^-3** | 0.95 | 1.89 |
| **GBM^50^-1** | 0.98 | 2.33 |
| **GBM^50^-2** | 0.97 | 2.17 |
| **GBM^50^-3** | 0.95 | 1.97 |
| **GBM^3^-1** | 0.96 | 1.88 |
| **GBM^3^-2** | 0.97 | 2.28 |
| **GBM^3^-3** | 0.98 | 2.23 |
| **GBM^0.2^-1** | 0.98 | 2.67 |
| **GBM^0.2^-2** | 0.97 | 2.39 |
| **GBM^0.2^-3** | 0.96 | 2.21 |
