# Supplementary material for: Modulation of mitochondrial DNA copy number in a model of glioblastoma induces changes to DNA methylation and gene expression of the nuclear genome in tumours
Source: Epigenetics Chromatin. 2018 Sep 12;11:53. doi: 10.1186/s13072-018-0223-z (PMC6136172; doi:10.1186/s13072-018-0223-z)
Supplement: Supplementary file 2 — Additional file 2. Summary of CNV regions identified in each cohort of tumours and overlapping with DMRs using the Nexus 9.0 software. [file 13072_2018_223_MOESM2_ESM.docx]

**Additional file 2 Summary of CNV regions identified in each cohort of tumours and overlapping with DMRs using the Nexus 9.0 software.**

To investigate the impact of CNV on the results, tumour samples were genotyped using the Illumina Global Screening array which covers over 700 K SNPs in the human genome. CN-gain and loss, and loss of heterozygosity (LOH) regions were identified in the tumour samples (Supplementary Table 1). The GBM^100^ tumours exhibited 13 CN-gain and 14 CN-loss regions (Supplementary Table 2). The GBM^3^ tumours possessed 10 regions of CN-gain and 7 regions of CN-loss. The GBM^50^ and the GBM^0.2^ tumours had 4 CN-gain regions and 3 CN-loss regions. Amongst the regions with CNVs, two of them were identified to overlap with the DMRs identified in the GBM^0.2^ tumours (p ≤ 0.001), none of the other CNV regions identified in the tumours overlapped with the DMRs. Nevertheless, no CNV was identified to be significantly different between the tumour groups, as assessed using the NEXUS Copy Number Module. This negates the potential effect of CNV on the analysis of MeDIP-Seq outcomes.

Furthermore, as the tumours were derived from the same parental cell line, each group of tumours had similar numbers of LOH: 460 in the GBM^100^ tumours, 461 in the GBM^50^ tumours, 471 in the GBM^3^ tumours, and 464 in the GBM^0.2^ tumours. By contrast to the regions with CN gain and loss, more than 99% of the regions with LOH were commonly identified amongst the tumours.

**Supplementary Table 1. CNV regions identified in each cohort of tumours determined using the Nexus 9.0 software.**

|  | **GBM^100^** | **GBM^50^** | **GBM^3^** | **GBM^0.2^** |
| --- | --- | --- | --- | --- |
| **CN GAIN** | chr1:0-832,780  chr1:150,990,558-151,341,916  chr1:3,205,527-3,282,778  chr2:15,821,548-15,950,064  chr2:166,859,135-166,868,616  chr2:166,915,083-166,915,196  chr4:3,266,069-3,487,234  chr7:54,723,018-55,297,327  chr8:146,179,817-146,364,022  chr15:63,352,476-63,354,438  chrX:155,119,599-155,270,560  chrX:18,528,151-18,593,560  chrX:32,360,246-32,473,852 | chr2:15,863,254-15,950,064  chr2:16,146,366-16,212,772  chr2:16,857,519-16,931,905  chrX:32,360,138-32,367,584 | chr1:797,934-843,780  chr11:2,389,848-2,448,378  chr11:2,604,703-2,606,505  chr12:195,412-252,925  chr13:115,090,132-115,090,198  chr14:107,188,511-107,349,540  chr18:77,949,664-78,077,248  chr21:48,052,620-48,129,895  chr3:0-185,862  chr7:159,084,752-159,138,663 | chr19:59,075,899-59,128,983  chr21:48,006,207-48,129,895  chr5:0-60,908  chr7:159,049,106-159,138,663 |
| **CN LOSS** | chr1:224,267,066-224,397,010  chr1:235,149,351-235,458,521  chr1:236,217,325-236,261,585  chr11:108,098,592-108,115,578  chr12:34,700,607-35,800,000  chr12:38,223,599-38,312,274  chr13:20,347,985-20,493,343  chr15:44,489,615-44,856,850  chr17:41,256,189-41,278,437  chr2:47,635,575-47,638,531  chr2:47,639,550-47,651,407  chr4:40,034,626-40,103,792  chr6:74,067,561-74,350,353  chr7:61,814,839-62,249,302 | chr1:235,861,367-235,914,143  chr12:34,700,607-35,800,000  chr5:45,947,493-46,399,093 | chr14:34,950,211-35,075,115  chr22:29,092,959-29,121,341  chr4:57,682,271-57,697,521  chr5:46,117,343-46,399,093  chr6:107,469,470-107,540,766  chr7:61,859,728-62,000,116  chr8:43,457,427-43,831,387 | chr10:42,502,256-42,632,033  chr11:50,392,461-51,328,556  chr12:34,507,145-35,800,000 |

**Supplementary Table 2. CNV analysis determined using the Nexus Copy Number Module.**

|  |  | CN gain | CN loss | LOH |
| --- | --- | --- | --- | --- |
| GBM^100^ | Total | 13 | 14 | 460 |
|  | Unique | 9 | 11 | 1 |
| GBM^50^ | Total | 4 | 3 | 461 |
|  | Unique | 1 | 0 | 2 |
| GBM^3^ | Total | 10 | 7 | 471 |
|  | Unique | 8 | 5 | 5 |
| GBM^0.2^ | Total | 4 | 3 | 464 |
|  | Unique | 2 | 2 | 3 |
