# Supplementary material for: Modulation of mitochondrial DNA copy number in a model of glioblastoma induces changes to DNA methylation and gene expression of the nuclear genome in tumours
Source: Epigenetics Chromatin. 2018 Sep 12;11:53. doi: 10.1186/s13072-018-0223-z (PMC6136172; doi:10.1186/s13072-018-0223-z)
Supplement: Supplementary file 3 — Additional file 3. Primer pairs for real time PCR. [file 13072_2018_223_MOESM3_ESM.docx]

**Additional file 3. Primer pairs for real time PCR.**

| **Human Gene** | **Forward primer (5’-3’)** | **Product size** | **Annealing Tm ( °C)** |
| --- | --- | --- | --- |
|  |  |  |  |
| β-globin-F | CAACTTCATCCACGTTCACC | 268 | 57 |
| β-globin-R | GAAGAGCCAAGGACAGGTAC |  |  |
| mtDNA-F | CGAAAGGACAAGAGAAATAAGG | 152 | 53 |
| mtDNA-R | CTGTAAAGTTTTAAGTTTTATGCG |  |  |
| SLC27A1-F | TCTCTCTGCTTCCCCAGGAT | 206 | 58 |
| SLC27A1-R | CGGATCAGCACAGAGAGACC |  |  |
| RHOT2-F | CAGAGCGAAAGGCTTGAGGA | 136 | 58 |
| RHOT2-R | ACAGGATCAGCGACGTCTTC |  |  |
| OGFR-F | CTCCGCATCACACGCATC | 207 | 58 |
| OGFR-R | AAGTGCTCCCAGGCGAA |  |  |
| MICALL2-F | ACATGATTGAGAAGCTGGGCCT | 108 | 58 |
| MICALL2-R | GCCCTACTGGCTACTACTGGG |  |  |
| MAF-F | AGCAAGTCGACCACCTCAAG | 168 | 58 |
| MAF-R | CTGGAATCGCGTGTCAGACT |  |  |
| L3MBTL1-F | GGTTTGGCTGGTGTAGCTTG | 179 | 58 |
| L3MBTL1-R | ACTCGCTTATCCGGAACTGG |  |  |
| KCNC1-F | CCCGTCATCGTGAACAATTTC | 150 | 58 |
| KCNC1-R | ACTGTGGTGTGGAGAGTTTAC |  |  |
| GPSM1-F | CCTTCTTTGAGGCTGCTGT | 135 | 58 |
| GPSM1-R | GGAGGTCATGCTTGTGGTATT |  |  |
| BAIAP2-F | AGAGCACACCCATCATGAAC | 145 | 58 |
| BAIAP2-R | GGGAGTGTGTTGGAGTAGGA |  |  |
