# Supplementary material for: Modulation of mitochondrial DNA copy number in a model of glioblastoma induces changes to DNA methylation and gene expression of the nuclear genome in tumours
Source: Epigenetics Chromatin. 2018 Sep 12;11:53. doi: 10.1186/s13072-018-0223-z (PMC6136172; doi:10.1186/s13072-018-0223-z)
Supplement: Supplementary file 5 — Additional file 5. Taqman assays used in the Fluidigm qPCR arrays. [file 13072_2018_223_MOESM5_ESM.docx]

**Additional file 5. Taqman assays used in the Fluidigm qPCR arrays.**

| **Gene name** | **Taqman assay number** |
| --- | --- |
| Actb | Hs99999903_m1 |
| 18SrRNA | Hs99999901_s1 |
| TWNK | Hs00958168_g1 |
| DNMT1 | Hs00154749_m1 |
| DNMT3A | Hs01027166_m1 |
| DNMT3B | Hs00171876_m1 |
| EGFR | Hs01076090_m1 |
| ESRRA | Hs01067166_g1 |
| ESRRB | Hs01584024_m1 |
| ESRRG | Hs00155006_m1 |
| HIF1a | Hs00153153_m1 |
| HPRT1 | Hs02800695_m1 |
| IDH1 | Hs01855675_s1 |
| IDH2 | Hs00158033_m1 |
| OAZ1 | Hs00427923_m1 |
| POLG | Hs00160298_m1 |
| POLG2 | Hs00200546_m1 |
| POLRMT | Hs04187596_g1 |
| PPARGC1A | Hs01016719_m1 |
| SIRT1 | Hs01009006_m1 |
| SIRT2 | Hs00247263_m1 |
| STAT3 | Hs01047580_m1 |
| TERT | Hs00972650_m1 |
| TET1 | Hs00286756_m1 |
| TET2 | Hs00325999_m1 |
| TET3 | Hs00379125_m1 |
| TFAM | Hs00273372_s1 |
| TFB2M | Hs00915025_m1 |
| TOP1MT | Hs01080056_m1 |
| TP53 | Hs01034249_m1 |
